# Multiple inferior vena cava aneurysms mimic a retroperitoneal tumor: a case report

**DOI:** 10.1186/s12894-020-00703-5

**Published:** 2020-09-01

**Authors:** Yuzhi Zuo, Zhenyu Zhang, Bingbing Shi, Zhigang Ji, Zhongming Huang

**Affiliations:** Department of Urology, Peking Union Medical College Hospital, Peking Union Medical College and Chinese Academy of Medical Sciences, No.1 Shuaifuyuan, Beijing, 100730 P.R. China

**Keywords:** Inferior vena cava, Diverticular aneurysm, Retroperitoneal laparoscopy, Case report

## Abstract

**Background:**

Inferior vena cava (IVC) aneurysms are extremely rare with variable clinical manifestations. Patients are usually asymptomatic or present with complications of thrombosis and rupture. To date, there have been only a few reports of the condition in the literature, and diagnosis of IVC aneurysms may be difficult.

**Case presentation:**

A 33-year-old male patient presented to hospital because of a retroperitoneal mass found by computerized tomography during a health examination. He was asymptomatic, and post medical history and physical examination were unremarkable. Laboratory tests including tests for paraganglioma were all negative. Contrast-enhanced computed tomography scan revealed a stenosis of IVC in the suprarenal segment and two retroperitoneal mass on the right side of IVC. The larger one is about 3 cm in diameter and the smaller one is about 1 cm in diameter, which was considered as a retroperitoneal tumor with an enlarged lymph node. However, two IVC diverticular aneurysms were confirmed during the retroperitoneal laparoscopic exploration. The larger aneurysm was resected from the IVC successfully. Since the smaller aneurysm was about 1 cm in diameter without thrombosis, we did not resect it during surgery. The patient recovered well from surgery and discharged from our department successfully.

**Conclusions:**

This is the first report of multiple IVC aneurysms. Because of the extremely low prevalence of IVC diverticular aneurysm, it may be misdiagnosed as other disease. Due to the high rate of thrombosis, surgical treatment especially retroperitoneal laparoscopy is recommended for small diverticular aneurysms.

## Background

Aneurysms are local vascular dilatations that develop when part of the vascular wall weakens, which mostly occur in arterial system. Although venous aneurysms can also occur throughout the body [1], they are relatively uncommon, and inferior vena cava (IVC) aneurysms are extremely rare. The first case of an IVC aneurysm was reported in 1972 by Conn [2]. To date, there have been only a few reports of the condition in the literature, and diagnosis of IVC aneurysms may be difficult. Here, we describe a case involving multiple IVC malformations, including aneurysms that mimicked a retroperitoneal tumor with an enlarged lymph node.

## Case presentation

**Fig. 1:**
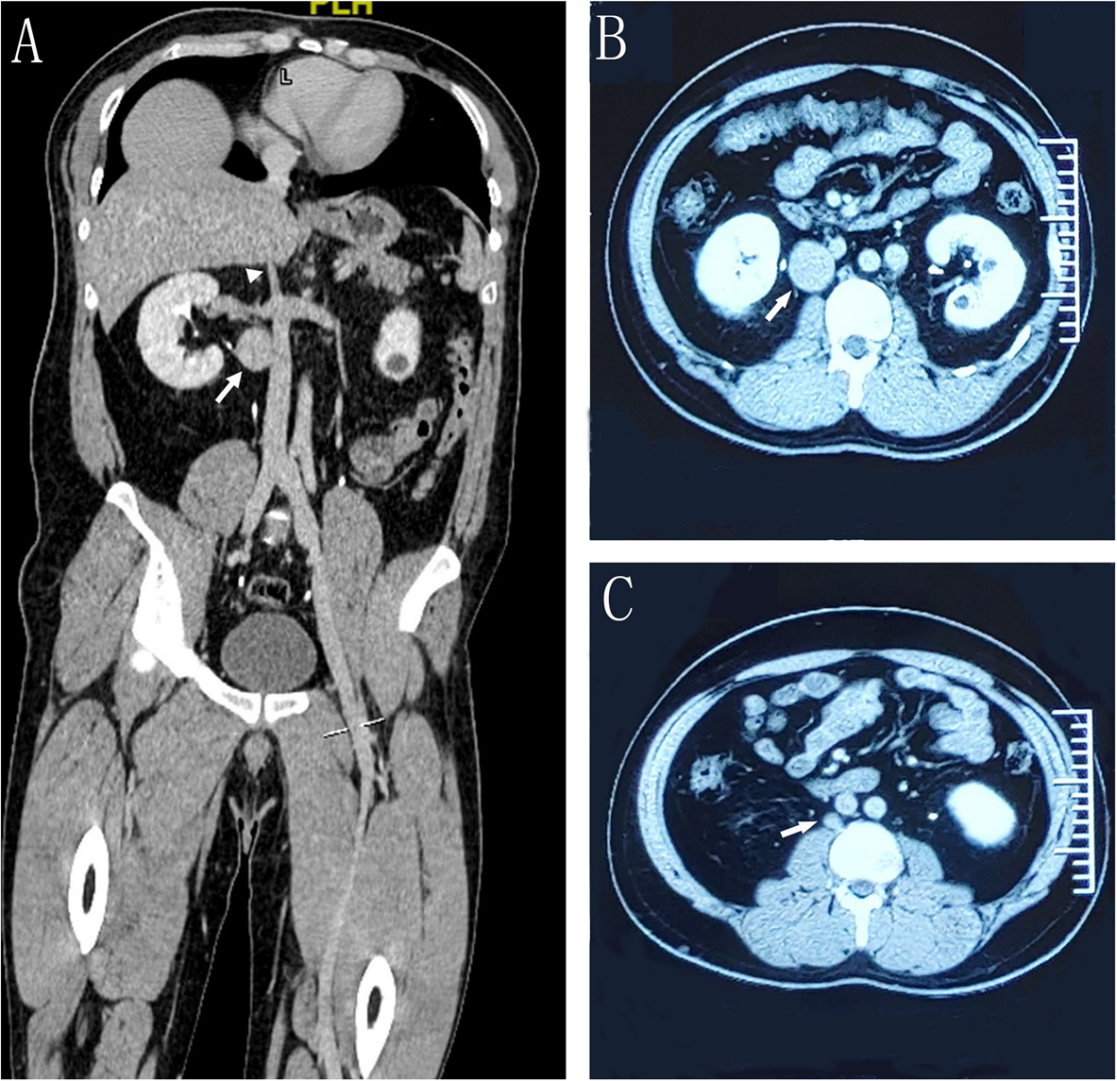
Contrast-enhanced CT imaging. **a** Coronal view showing the stenosis of IVC (arrowhead) and a retroperitoneal mass on the right side of the infrarenal IVC (long arrow). **b** and **c** Axial view showing the two retroperitoneal mass (long arrow)

A 33-year-old Chinese male patient presented to Urology Department because of a retroperitoneal mass that was incidentally found by computerized tomography (CT) during a health examination. He was asymptomatic and had no abdominal or back pain, episodic hypertension, palpitation, or lower limb edema. Post medical history and physical examination were unremarkable. Laboratory tests including complete blood count, liver and renal function and D-Dimer were normal. Tests for paraganglioma such as 24-h urinary catecholamine and somatostatin receptor imaging were negative. Contrast-enhanced CT scan revealed a stenosis of IVC in the suprarenal segment (Fig. 1a), a 34 mm × 30 mm × 33 mm retroperitoneal mass on the right side of the infrarenal IVC (Fig. 1a and b) and a small mass about 10 mm in diameter in the retroperitoneal area that was considered an enlarged lymph node (Fig. 1c). No thrombosis was found in the IVC. Therefore, a diagnosis of retroperitoneal tumor with possible lymph node metastasis was considered.

**Fig. 2:**
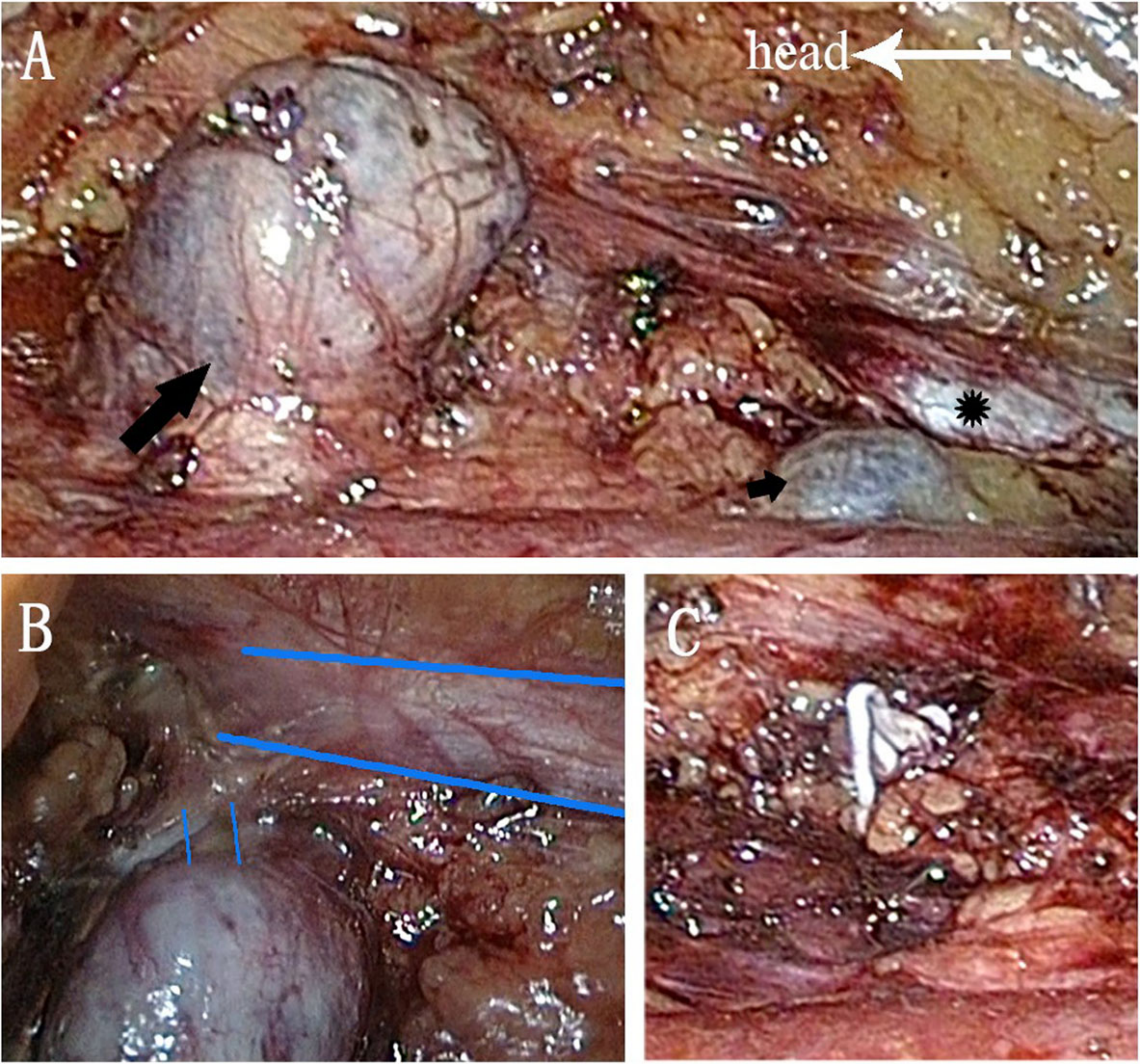
Intra-operative photographs. **a** IVC (black star) and two aneurysms (black long arrow and short arrow). **b** A narrow neck between the larger aneurysm and IVC (blue thick lines showing edges of IVC and thin lines showing the neck of the aneurysm). **c** Hem-o-lok in the neck of aneurysm after resection

To further confirm our diagnosis, a retroperitoneal laparoscopic exploration was performed. During the surgery, we found that both mass were diverticular aneurysms of IVC (Fig. 2a). There was a very narrow neck between the larger aneurysm and IVC (Fig. 2b). A hem-o-lok clamp was applied on the neck of aneurysm (Fig. 2c), and the aneurysm was resected from the IVC successfully. Since the smaller aneurysm was about 1 cm in diameter without thrombosis, we did not resect it during surgery. However, the stenosis of IVC may cause venous hypertension which can be a risk factor for aneurysms progression. Therefore, we referred the patient to Vascular Surgery Department for pressure gradient test across the suprarenal IVC to determine the following therapy. They suggested to monitor the size of aneurysm annually to determine further treatment. The patient recovered well from surgery and discharged from our department successfully. The pathology shows vascular wall tissue with a lot fibrous tissue, which is consistent with the diagnosis of IVC aneurysm. However, there is no intact vascular wall identified.

## Discussion and conclusions

Our patient had multiple IVC malformations, including two IVC aneurysms. IVC aneurysms are extremely rare with variable clinical manifestations. They may be discovered incidentally in asymptomatic patients, as it was in our case, or may present with complications, such as leg swelling, pulmonary embolism and retroperitoneal mass related effects, like abdominal/low back pain, hydronephrosis and bowel obstruction [3–5]. Because the symptoms are non-specific, imaging tests like ultrasonography, contrast-enhanced CT, magnetic resonance imaging and cavography play a vital role in the diagnosis of IVC aneurysms [6]. However, some IVC aneurysms could be confused with retroperitoneal tumors, such as renal carcinoma, sarcomas, enlarged lymph nodes, neurogenic tumors and primary IVC tumors [7–9], especially when the lumen of the aneurysm is completely thrombosed [8]. Therefore, comprehensive consideration of various imaging tests results can increase the accuracy of diagnosis. Cavography or surgical exploration is recommended when an IVC aneurysm is highly suspected. Biopsy should be more carefully chosen when the nature of the mass is unclear.

Pathological studies have revealed that IVC aneurysms are composed of all three layers of the normal venous wall with thinning elastic and muscular layers [10]. However, the etiology of IVC aneurysms is still unclear. Trauma, injury, inflammation, longstanding venous hypertension secondary to heart failure, cardiomyopathy, tricuspid valve lesions, constrictive pericarditis and stenosis of IVC are all risk factors of IVC aneurysms. As many cases are diagnosed in childhood, congenital defects like embryological venous malformations are considered the most common cause [11–15].

Embryological development of IVC is complex. Through complex sequential processes of development, anastomosis and regression of three parallel veins (the postcardinal, subcardinal and supracardinal veins), the mature IVC forms [16, 17]. The normal IVC is composed of four segments, including the infrarenal, renal, suprarenal and hepatic IVC, which are derived from supracardinal veins, subcardinal-supracardinal vein anastomoses, subcardinal veins and subcardinal-hepatic vein anastomoses, respectively [18, 19]. Our patient presented with no risk factors, and multiple IVC malformations were observed, indicating that an embryological development anomaly may be the cause of his condition.

In the literature, there are two classification systems for IVC aneurysms (Table 1). The Thompson and Lindenauer classification includes three types based on the etiology of aneurysm [20]. However, application of this classification is limited, since the etiology of aneurysm is unclear in most cases. Gradman and Steinberg classified IVC aneurysms into four types depending on the relation to the hepatic vein and the resultant obstruction [21]. Type I IVC aneurysms were most common, followed by type III. Type IV IVC aneurysms were rare [12]. This classification has more utility in clinic and is used in our article. However, both of the classification systems have limited guidance for treatment and prognosis.

**Table 1:** IVC aneurysm classification system

	Gradman and Steinberg classification	Thompson and Lindenauer classification
Type I	Aneurysms of the suprahepatic IVC without venous obstruction	Congenital aneurysm
Type II	Aneurysms associated with interruption of the IVC above or below the hepatic vein	Acquired aneurysm
Type III	Aneurysms confined to the infrarenal IVC without associated venous anomaly	Aneurysm secondary to arteriovenous fistula
Type IV	Miscellaneous	

Due to potential life-threatening morbidities, such as thromboembolic events and rupture, treatment of IVC aneurysms is recommended [3, 12, 22]. However, there is no consensus on patient management. In some literature reviews, surgical treatment such as resection and reconstruction is recommended for types II–IV or symptomatic patients [3, 11, 12]. Endovascular techniques were also recently reported in the treatment of IVC aneurysm. Michel et al. [23] successfully embolized a congenital large saccular aneurysm of the infrarenal IVC in a 2.5-year-old male with coils and an Amplatzer vascular plug device. Falkowski et al. [24] implanted a custom-made stent-graft in an infrarenal IVC aneurysm to exclude it from the circulation completely. Walsh et al. [25] performed balloon angioplasty to improve the congenital stenosis of IVC in a type II aneurysm, and aneurysm size was decreased during the follow-up. Asymptomatic type I IVC aneurysm can be managed conservatively by regularly monitoring aneurysm size and the development of any complications [26]. Medical management can include anticoagulation and IVC filter placement in patients with thrombosis.

In our case, it was difficult to classify the IVC aneurysms accurately. There were two infrarenal IVC aneurysm and stenosis of suprarenal IVC. It was unclear whether the aneurysm was associated with the stenosis of IVC. In our opinion, there was no obvious dilation of the distal IVC, and the aneurysms were relatively far from the stenosis. Therefore, the infrarenal IVC aneurysms were probably not associated with the stenosis, and thus, are type III IVC aneurysms. IVC aneurysms can be saccular, fusiform or diverticular. The diverticular type is rare, and to date there have been only eight cases reported in the literature [3, 16, 21, 22, 27–30] (Table 2). All reported patients were male, and all aneurysms were located at the infrarenal IVC segment, which is different from the IVC aneurysm entity. Therefore, diverticular IVC aneurysms may be a special type of IVC aneurysm, and it is probably caused by the incomplete regression of supracardinal vein branches. Due to the high rate of thrombosis associated with diverticular IVC aneurysms, surgical treatment is recommended. Compared to open surgical operation, retroperitoneal laparoscopy is less invasive with a shorter in-hospital duration and less blood loss, and it is especially suitable for treating small aneurysm. Endovascular treatment is an alternative method for patients without thrombosis.

**Table 2:** Reported diverticular IVC aneurysms

Author	Year	Age (year)	Gender	Presentation	Location	Thrombosis	Treatment	Outcome
Hasan, et al. [16]	1992	59	Male	Abdominal pain	Infrarenal	No	Open surgical resection	Good
Levesque, et al. [27]	1993	70	Male	Asymptomatic	Infrarenal	Yes	Monitoring	No changes in thrombosis or aneurysm size
Gradman, et al. [21]	1993	45	Male	Back and chest pain, swelling of left lower extremity	Infrarenal	Yes	Open surgical resection	Good
Davidovic, et al. [3]	2008	27	Male	Abdominal pain and swelling of bilateral lower extremities	Infrarenal	Yes	Open surgical resection	Good
Deshpande, et al. [28]	2010	40	Male	Bilateral lower extremity swelling and pain	Infrarenal	Yes	Open surgical resection and anticoagulation	Good
Weber, et al. [29]	2011	13	Male	Left lower extremity swelling and pain	Infrarenal	Yes	Open surgical resection and anticoagulation	Good
Tadayon, et al. [22]	2019	22	Male	Abdominal pain	Infrarenal	No	Open surgical resection	Good
Ladurner, et al. [30]	2019	23	Male	NM	Infrarenal	Yes	Anticoagulation	NM

*NM* Not mentioned

Table 2. Reported diverticular IVC aneurysms (in the end of the file).

IVC aneurysms are extremely rare, and this is the first report of multiple IVC diverticular aneurysms. It should be a differential diagnosis in the evaluation of retroperitoneal tumors. In addition, IVC diverticular aneurysms may be a special type. Due to the high rate of thrombosis, surgical treatment is recommended and retroperitoneal laparoscopy is suitable for small aneurysms.

## Data Availability

All data generated or analyzed during this study are included in this published article.

## Abbreviations

IVC: Inferior vena cava
CT: Computerized tomography
